# Concomitant *JAK2* V617F-positive polycythemia vera and *BCR-ABL*-positive chronic myelogenous leukemia treated with ruxolitinib and dasatinib

**DOI:** 10.1038/bcj.2015.77

**Published:** 2015-10-02

**Authors:** A Zhou, E M Knoche, E K Engle, D A C Fisher, S T Oh

**Affiliations:** 1 Division of Hematology, Washington University School of Medicine, St Louis, MO, USA

Myeloproliferative neoplasms (MPNs) are myeloid malignancies characterized by stem cell-derived clonal myeloproliferation. There are seven designated conditions under the category of MPNs including chronic myelogenous leukemia (CML) and polycythemia vera (PV).^1^ CML is characterized by the presence of the Philadelphia chromosome (Ph), a translocation involving chromosomes 9 and 22 that results in the formation of the *BCR-ABL* fusion gene. PV is a Ph-negative MPN characterized by erythrocytosis and the presence of an activating *JAK2* mutation in >95% of cases. PV and CML are considered to be mutually exclusive; however, rare cases of concurrent Ph-positive CML and a *JAK2* V617F-positive PV have been described in the literature.^2, 3, 4, 5, 6, 7, 8, 9^ Previous reports have suggested that the two disorders may arise independently,^2, 3, 4^ or from within the same clone,^5, 6, 7, 8, 9^ but definitive clonal analysis has never been documented. We report a case of a patient with *JAK2* V617F-positive PV who subsequently developed chronic phase Ph-positive CML. The patient was successfully treated with the combination of dasatinib and ruxolitinib. Cytogenetic and molecular responses suggested that the two disorders arose independently, which was corroborated by genotyping analyses of single-cell-derived colonies.

A 55-year-old woman was first diagnosed with PV in 2001 when she presented with a hemoglobin (Hgb) of 20.0 g/dl. Over the ensuing 10 years, the patient was treated with aspirin and intermittent hydroxyurea and anagrelide, with poor compliance. In May 2011 (10 years after initial PV diagnosis), she presented with worsening fatigue, weight loss and splenomegaly. Laboratory studies revealed an elevated white blood cell count (WBC) of 45 × 10^9^/l and a Hgb of 9.5 g/dl. Examination of the peripheral smear demonstrated leukoerythroblastic changes with few circulating blasts. A bone marrow biopsy showed hypercellularity with granulocytic and megakaryocytic hyperplasia, no excess blasts, and moderate fibrosis on reticulin staining. A diagnosis of post-PV myelofibrosis was initially suspected; however, cytogenetics showed t(9;22) in 20 out of 20 metaphase cells and fluorescence *in situ* hybridization for *BCR-ABL* was positive (92.5%). Quantitative PCR for *JAK2* V617F revealed an allele burden of 6%. Therefore, it appeared that the patient had developed CML with relative clonal dominance over PV.

Treatment with dasatinib 100 mg daily was initiated in July 2011, resulting in dramatic improvement in the patient's leukocytosis and anemia within 1 month (Figure 1a). Shortly thereafter, however, her WBC and platelet count began to rise. Three months after starting dasatinib, the patient's complete blood count (CBC) showed a WBC of 42 × 10^9^/l, Hgb 12.3 g/dl and platelet count of 799 × 10^9^/l. *BCR-ABL* by fluorescence *in situ* hybridization had decreased to 4.0% however, the *JAK2 V617F* allele burden had increased to 83% (Figure 1b). Therefore, recrudescence of leukocytosis and thrombocytosis in this patient appeared to be due to re-emergence of the *JAK2*-mutant clone in the setting of suppression of the *BCR-ABL*-positive CML clone by dasatinib.

The patient was treated with hydroxyurea without significant improvement in leukocytosis or thrombocytosis, and there was also no response when hydroxyurea was changed to anagrelide. A repeat bone marrow biopsy performed in May 2012 (300 days after initiation of dasatinib) revealed a hypercellular marrow, no increased blasts and severe fibrosis on reticulin staining. Cytogenetic analysis demonstrated a complete cytogenetic response with *BCR-ABL* by qRT-PCR detected at 0.001% (normalized to β-2 microglobulin). Despite minimal residual clonal burden from CML, the patient continued to report fatigue and poor appetite, progressive weight loss of 15 pounds and persistent splenomegaly.

Owing to the progressive increase in counts, ongoing constitutional symptoms and poor tolerance of hydroxyurea, treatment with ruxolitinib 10 mg twice daily was commenced (in addition to continuation of dasatinib) in June 2012. Within 6 weeks, there was significant improvement in the patient's blood counts (WBC 11.9 × 10^9^/l, Hgb 11.9 g/dl, platelets 297 × 10^9^/l), as well as improvement in constitutional symptoms and splenomegaly. The patient tolerated treatment with the combination of ruxolitinib and dasatinib well, but was noted to have worsening anemia in September 2012, approximately 4 months after starting treatment with both therapies. She was advised to hold ruxolitinib but accidentally held dasatinib for 1 month. Subsequently, dasatinib was resumed and ruxolitinib was held in October 2012 for a month, following which the patient's Hgb improved, and she was restarted on ruxolitinib at a decreased dose of 5 mg twice daily. Two months later, the dose of ruxolitinib was increased to 10 mg alternating with 5 mg twice daily.

Three years after starting treatment with dasatinib and ruxolitinib, the patient's symptoms and CBC remain stable and she has continued on combination therapy with dasatinib and ruxolitinib. The patient has maintained a complete cytogenetic response with low level *BCR-ABL* detectable by qRT-PCR, while the *JAK2* V617F allele burden has fluctuated between 43 and 96%.

Serial quantitative measurements of *BCR-ABL* and *JAK2* V617F following treatment with dasatinib demonstrated a marked decrease in *BCR-ABL* levels with a concomitant increase in the *JAK2* V617F allele burden, suggesting that the two disorders arose from independent clones. To further address this issue, single cell-derived progenitor colonies were isolated and genotyped for *BCR-ABL* and *JAK2* V617F (Figure 2). The majority of the colonies demonstrated only the presence of *JAK2* V617F or *BCR-ABL*, but not both, confirming that the two disorders arose within distinct clones.

Several other case reports observed a similar increase in the *JAK2* V617F allele burden following successful treatment with an *ABL* tyrosine kinase inhibitor (TKI),^5, 6, 7, 8^ including a recently published report of two patients with PV/CML who were treated with the combination of imatinib and ruxolitinib.^9^ Similar to our case, treatment with imatinib did not lead to improvement in constitutional symptoms or splenomegaly in either patient, although they both achieved complete cytogenetic and major molecular responses of their CML. The addition of ruxolitinib to imatinib in both cases produced an improvement in constitutional symptoms and splenomegaly; however, both patients required dose reductions of ruxolitinib and imatinib owing to hematologic toxicity.

To our knowledge, this is the first case report of a patient with concurrent PV and CML treated with a JAK inhibitor and a second generation ABL TKI that definitively demonstrates that the two neoplasms arose from separate clones. None of the prior case reports included correlative clonal analysis of patients treated with this combination of therapy. Genotyping of individual progenitor colonies suggests that the two disorders arose from independent clones, which is consistent with the findings from serial quantitative measurements of *BCR-ABL* and *JAK2* V617F during treatment; however, it is still possible that these two clones originated from a shared ancestral clone.

The combination of ruxolitinib and dasatinib was safe and effective in the treatment of concomitant PV and CML in this patient. The optimal schedule of treatment (for example, simultaneous, sequential or syncopated TKI regimens) for treatment of patients with concomitant PV/CML is still unclear, but can potentially be guided by molecular monitoring and biologic correlative analyses. Ultimately, the best treatment approach will be determined by the kinetic dynamics of each clone and the tolerability of combining TKIs.

## Figures and Tables

**Figure 1:**
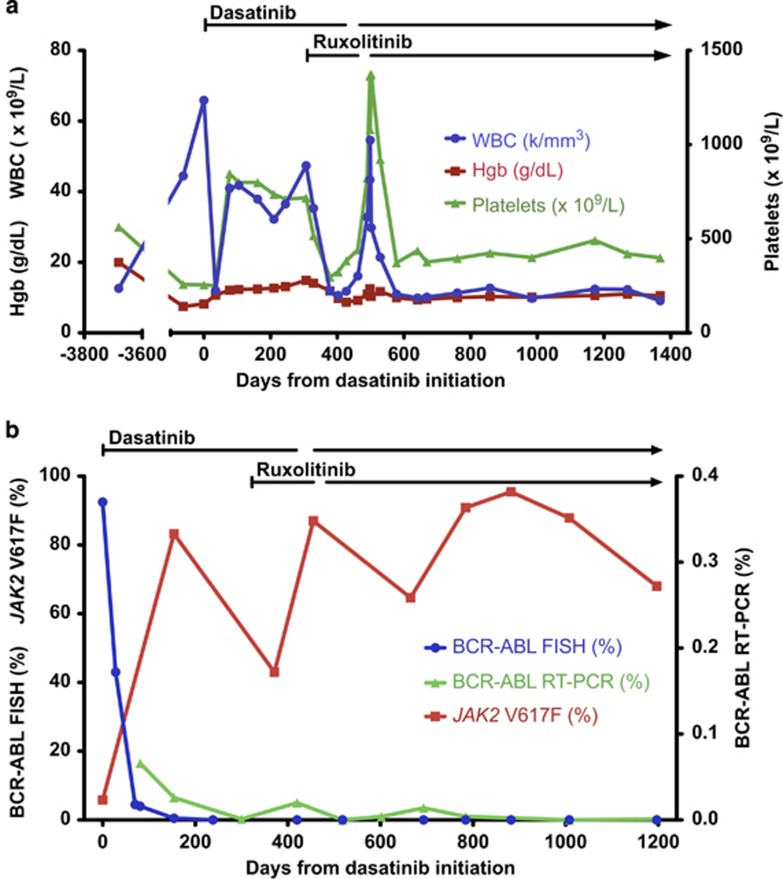
Hematologic, cytogenetic and molecular data. (**a**) CBC parameters over time are shown in relationship to treatment with dasatinib and ruxolitinib. (**b**) Cytogenetic and molecular analyses of *BCR-ABL* and *JAK2* V617F over time are shown in relationship to treatment with dasatinib and ruxolitinib.

**Figure 2:**
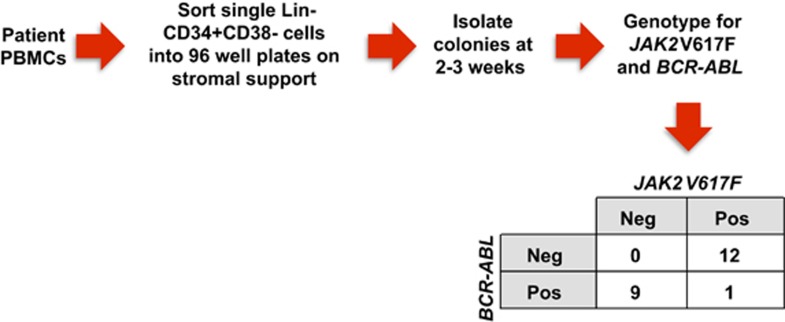
Genotyping of individual progenitor colonies. Informed consent was obtained from the patient, and samples were collected under a protocol approved by the Washington University Human Studies Committee (#01-1014). Colonies were isolated from the peripheral blood at time of CML diagnosis and were genotyped for *BCR-ABL* and *JAK2* V617F. The number of colonies with each genotype is shown.
